# Two Cases of Rupture of the Uterus—Recovery of One

**Published:** 1847-03

**Authors:** 


*Two Cases of Rupture of the Uterus—Recovery of One.*—M. Robiquet was called to attend, in labour, a female, ætat. 32, who had previously had one child, and had never suffered under any symptoms of uterine disease; the present, her second pregnancy, had gone on favourably until about two hours before M. R.'s visit, when during a strong uterine contraction she felt something suddenly give way within her. It seemed to her as if her bowels had been torn, but soon an apparent calm succeeded this painful sensation.

On M. R.'s entrance he found the patient's countenance flushed, her skin moist, pulse 90, small and thready, respiration slow but regular; she had acute pain in the abdomen, with the sensation as of a weight rolling about in the middle of the belly and crushing the intestines, the uterine contractions were few and transient. The abdomen, depressed and irregular, had lost its rounded form, and permitted the limbs of the fœtus to be distinctly felt, and easily laid hold of through its parietes; and the child swayed from right to left, according to the movements of the patient. On vaginal examination, the head of the fœtus was felt at the uterine orifice. This latter circumstance induced M. R. to wait a short time, but finding that the powers of the mother were being rapidly exhausted, he applied forceps and extracted a female child, which lived only a few minutes. Scarcely was the fœtus expelled, when a soft rose-coloured slightly inflamed mass projected from the vagina and hung down between the thighs of the patient; this was at once recognized as part of the small intestine and the epiploon. M. R. at once returned the intestinal mass into the vagina, and gently pushed it onward to the fundus of the uterus, in which, rather to the right side of that organ, he found an opening large enough to admit his hand to pass through easily. Having at length with some difficulty returned the whole intestinal mass through the wound, he passed his right hand through the laceration so as to cover its aperture, while with his left, externally, he used frictions over the hypogastric region, with a view of inducing uterine contraction, and for the same object fifteen grains of ergot were administered. After a lapse of two or three minutes, M. R. felt the edges of the wound to approach, and the body of the uterus to make some efforts at contraction. After a second contraction had considerably diminished the diameter of the laceration, M. R. gently drew his hand out of the wound into the cavity of the uterus, and applied it to the inner surface of the rupture, to prevent any projection of the intestines through the opening. A second dose of ergot had been previously administered, and in a short time the uterus contracted so powerfully that he was compelled to withdraw his hand from its cavity. A dangerous attack of metro-peritonitis followed, which was successfully treated in the usual manner. Symptoms of inflammation of the uterus continuing on the second day after delivery, M. R. introduced two fingers into its cavity, and finding a small knuckle of intestine protruding, he successfully effected its reduction. From this time the patient gradually improved, and was at length restored to complete health.

*Second Case.*—The following recently came under the notice of M. Dubois at the *Clinique d'Accouchements*:—The patient, ætat. 32, arrived at the termination of her third pregnancy, without any unfavourable symptoms. On the 9th June labour set in with pains, feeble, and recurring at long intervals. On the following day the membranes ruptured, accompanied with trifling hemorrhage; the labour pains ceased, and were replaced by continued and intermitting pain. The medical attendants, being ignorant of the true cause of the cessation of uterine contractions, administered ergot, in order to restore them. At noon of the same day the os uteri was completely dilated, the fœtal head resting at the brim, but no attempt was made at artificial delivery; at six in the evening she was carried to the *Clinique* in the following condition:—There was general lividity of the surface of the body; the features were contracted, and the radial pulse imperceptible. M. Dubois was called, and at first supposed that the patient laboured under cholera; but, having heard an account of the case, immediately recognized it as an example of rupture of the uterus. On applying the stethoscope the child was found to be dead; forceps might have been applied, but M. Dubois declined to interfere under the circumstances of the case, on the ground that any interference would have hastened the patient's dissolution, and would uselessly compromise the obstetrical art in the eyes of the public, who are always disposed to judge wrongly: the patient died in a few minutes after. On examining the body after death, an extensive rent was found in the right side of the uterus, reaching from the orifice of the organ to the round ligament, passing through the entire thickness of the substance of the uterus, but leaving its peritoneal tunic quite uninjured. In both of the preceding cases the rupture was spontaneous, and not the result of ill-directed mechanical interference. We must therefore admit, says the reporter of the cases, an organic predisposition to laceration of the uterus, which is probably nothing else than an inflammatory softening of its tissue. In both of the cases, too, the rupture was in the right side of the uterus, instead of in its anterior surface, as it is more generally observed. We agree with the reporter, in thinking that M. Dubois' motive for non-interference, viz. "a fear of public opinion, which is always wrong," is, more especially in one of his reputation and experience, a very ridiculous, and possibly a highly dangerous, guide in practice.—*Monthly Jour. of Med. Science, from Journ de Med. et de Chirurg.*

